# Secure LoRa Drone-to-Drone Communication for Public Blockchain-Based UAV Traffic Management

**DOI:** 10.3390/s25165087

**Published:** 2025-08-15

**Authors:** Jing Huey Khor, Michail Sidorov, Melissa Jia Ying Chong

**Affiliations:** 1 Connected Intelligence Group, University of Southampton Malaysia, Iskandar Puteri 79100, Johor, Malaysia; j.khor@soton.ac.uk (J.H.K.); m.j.y.chong@soton.ac.uk (M.J.Y.C.); 2 School of Electrical and Electronic Engineering, University of Southampton Malaysia, Iskandar Puteri 79100, Johor, Malaysia

**Keywords:** blockchain, D2D, protocol, UAV, UTM

## Abstract

**Highlights:**

**What are the main findings?**

- Efficient Drone-to-Drone communication protocol fusion with a blockchain network for the purpose of secure and efficient Unmanned Aerial Vehicle Traffic Management is presented.

**What is the implication of the main finding?**

- Superior performance compared to state of the art.
- Lower computational and storage costs.

**Abstract:**

Unmanned Aerial Vehicles (UAVs) face collision risks due to Beyond Visual Line of Sight operations. Therefore, UAV Traffic Management (UTM) systems are used to manage and monitor UAV flight paths. However, centralized UTM systems are susceptible to various security attacks and are inefficient in managing flight data from different service providers. It further fails to provide low-latency communication required for UAV real-time operations. Thus, this paper proposes to integrate Drone-to-Drone (D2D) communication protocol into a secure public blockchain-based UTM system to enable direct communication between UAVs for efficient collision avoidance. The D2D protocol is designed using SHA256 hash function and bitwise XOR operations. A proof of concept has been built to verify that the UTM system is secure by enabling authorized service providers to view sensitive flight data only using legitimate secret keys. The security of the protocol has been analyzed and has been proven to be secure from key disclosure, adversary-in-the-middle, replay, and tracking attacks. Its performance has been evaluated and is proven to outperform existing studies by having the lowest computation cost of 0.01 ms and storage costs of 544–800 bits.

## 1. Introduction

Beyond Visual Line of Sight (BVLOS) operation of Unmanned Aerial Vehicles (UAVs), i.e., operation conducted outside of the direct visual range of the pilot, significantly enhances the capabilities of UAVs by allowing them to operate and perform tasks well beyond the direct line of sight of the operator. This, however, introduces additional challenges. BVLOS flights pose collision risks due to the lack of direct visual guidance and increased vulnerability to environmental factors such as adverse weather, rogue drones, birds, etc. To address these risks, researchers have integrated high-speed cameras [1], LiDAR [2], radar systems [3], and radio frequency-based methods [4] for detecting obstacles and potentially malicious drones. However, effective UAV traffic monitoring goes beyond local sensing and requires coordinated tracking and information sharing among UAVs.

To enable safe and efficient UAV operations, a robust UAV Traffic Management (UTM) system is essential. Remote Identification (RID) plays a crucial role in this framework by enabling authorities and the public to identify UAVs and access flight data in real-time. While RID improves transparency, it also introduces serious privacy and security risks by making UAV identities and locations publicly visible [4,5]. Furthermore, current UTMs are typically centralized, making them susceptible to single points of failure, data tampering, and unauthorized access. These systems are unable to ensure secure and efficient interactions between service providers and end-users.

As a solution, researchers have proposed integrating blockchain technology into UTMs [6,7,8,9]. The decentralized and immutable nature of blockchain makes it a promising solution for enhancing security and trust in UAV ecosystems. Smart contracts can further automate critical tasks related to traffic monitoring, flight planning, and authorization. However, while blockchain enhances transparency, it also poses privacy risks, as sensitive flight data becomes permanently visible on the public ledger. Additionally, blockchain platforms such as Ethereum suffer from transaction latency (e.g., 6–45 s) [10], which limits their ability to support real-time UAV coordination, particularly in collision avoidance scenarios.

Therefore, Drone-to-Drone (D2D) communication emerges as a vital mechanism for real-time data exchange and coordination in low-altitude airspace [11]. D2D communication enables UAVs to autonomously negotiate flight paths and avoid collisions without relying on centralized infrastructure. Various technologies have been employed for D2D communication, including short-range wireless options such as Wi-Fi, Zigbee, and Bluetooth, as well as long-range alternatives like cellular and satellite links [12,13]. Among these, LoRa stands out due to its long transmission range, low power consumption, and operation in unlicensed Industrial, Scientific, and Medical (ISM) bands, making it suitable for energy-constrained UAVs operating in wide-area BVLOS missions [14].

LoRa and the MAVLink protocol have been widely adopted for telemetry and communication in UAV systems, where MAVLink structures the data and LoRa transmits it over long distances with minimal power consumption. However, MAVLink lacks built-in encryption mechanisms [15], leaving UAVs vulnerable to spoofing, interception, and other security attacks. Therefore, there is a need for a secure, low-latency D2D communication protocol tailored for UAVs operating in blockchain-based UTM environments.

Currently, no existing protocol enables UAVs from different service providers to perform D2D communication securely within a public blockchain-based UTM. To address this gap, this research proposes a lightweight LoRa-based D2D communication protocol that leverages a public blockchain to enable efficient flight management across multiple service providers, thereby minimizing latency and complexity in data exchange between UAVs. LoRa is adopted not only for its low power and wide range technical advantages, but also to support infrastructure independence for blockchain-based UTM systems.

This work focuses on the design of the communication protocol and its robustness against four common security threats frequently observed in Internet of Things (IoT) systems: key disclosure, adversary-in-the-middle, replay, and tracking attacks [16]. Specifically, the protocol utilizes session-based keys to protect against key disclosure, employs mutual authentication steps to counter adversary-in-the-middle attacks, incorporates timestamps and random number mechanisms to prevent replay attacks, and implements dynamic identity protection to mitigate tracking threats. It is essential to note that this paper does not address broader performance evaluation of LoRa, such as its susceptibility to noise interference, energy consumption, and transmission reliability. The contributions of this paper are as follows:

1. A lightweight protocol designed to enable secure D2D LoRa-based communication for secure public blockchain-based traffic management to avoid collisions.
2. This protocol enables UAVs to authenticate nearby UAVs from different service providers that are registered with the UTM system.
3. The protocol protects sensitive flight data of UAVs stored on the public blockchain-based UTM from attackers.
4. The proposed protocol has been analyzed to ensure it is secure from key disclosure, adversary-in-the-middle, replay, and tracking attacks.

The remainder of this paper is structured as follows: Section 2 reviews related work on improving the UTM systems using blockchain technologies, and the security of LoRa in UAV communication. Section 3 describes a secure LoRa D2D communication protocol for public blockchain-based traffic management systems. Section 4 presents a proof of concept for the proposed LoRa D2D communication protocol, utilizing a public blockchain-based UTM. Section 5 demonstrates the security of the proposed protocol, and Section 6 presents a comparative performance analysis. Section 7 describes the limitations of the system and how it can be addressed in future work. Section 8 concludes this paper.

## 2. Related Works

Blockchain has been successfully integrated with UAV networks to secure UAV communication and enable data protection (e.g., data integrity and identity authentication). It was further utilized with UTM systems, as demonstrated by Allouch et al., where a lightweight, permissioned blockchain-based UTM was employed to provide secure and immutable traffic data for UAV stakeholders, including UAV service providers, UAV operators, and end-users [7]. UAV data were stored in the InterPlanetary File System (IPFS), and the hashes generated from the data were stored on the HyperLedger Fabric blockchain. Rahman et al. proposed a blockchain-based policy enforcement mechanism in drone-based delivery service systems [6]. The mechanism establishes policies to allocate predefined flight paths for different drones at different times, thereby avoiding collisions and ensuring the privacy of citizens by restricting their access to unauthorized areas. The blockchain enforced policies to monitor compliance with drone flights and identify non-compliant drone services, penalizing the corresponding service providers.

Although blockchain is able to improve UAV network security, messages transmitted using communication channels such as Wi-Fi, cellular, and LoRa are vulnerable to security attacks. LoRaWAN, acting as a MAC layer for LoRa communication, offers robust security features. However, LoRaWAN can experience increased data latency and a higher rate of packet collision in densely populated networks due to its ALOHA-based access scheme [17]. One of the key contributors to latency in LoRaWAN is the Time on Air (ToA), which refers to the duration a packet occupies the wireless channel during transmission, determined by the Spreading Factor (SF) parameter. LoRaWAN with a lower SF (e.g., SF_7_ and SF_8_) has shorter airtime, typically below 700 ms; however, this rises to above one second for long-distance transmission using higher SFs (e.g., SF_9_–SF_12_) [18]. To address latency concerns, particularly for time-critical transmissions, Downlink Control Packets (DCPs) have been used where gateways periodically transmit DCPs to synchronize end devices, enabling them to schedule uplink transmissions [19]. Since high data latency and packet collision rates can affect the reliability of D2D communication, LoRa has been used in UAVs due to its low power consumption and lower latency compared to LoRaWAN [20]. Consequently, standard LoRaWAN is excluded from our comparative analysis as its architecture does not meet the real-time requirements of direct D2D communication among UAVs.

Despite its popularity, LoRa lacks security features, making it necessary to implement additional protection when utilizing it for communication. In contrast, the MAVLink protocol has been widely used in UAV networks due to its security features and lightweight structure, which is supported by different types of transport layers. With MAVLink signing capability, transmitted messages can be authenticated, ensuring their integrity and origin. This added security measure helps protect against tampering and unauthorized access, making the combination of MAVLink and LoRa a more secure choice for UAV communication [14]. However, MAVLink itself is susceptible to security attacks, such as adversary-in-the-middle and tracking attacks, because it does not support encryption [15]. Therefore, encryption techniques such as symmetric encryption algorithms have been widely employed to enhance the security of the MAVLink protocol [21,22]. Khan et al. utilized the Caesar cipher to encrypt MAVLink messages [15]. However, this cipher has been proven to be vulnerable to security attacks [23].

In addition to improving MAVLink security, several researchers have proposed secure protocols to enable reliable D2D communication [8,24,25,26]. However, existing research in the field lacks a specific focus on enhancing secure message transmission using LoRa-based D2D communication for secure public blockchain-based traffic management. Therefore, this paper aims to address this research gap.

## 3. Secure LoRa Drone-to-Drone Communication Using Public Blockchain-Based Traffic Management System

The proposed LoRa D2D communication protocol aims to support all UAVs offered by different service providers. Hence, in order to be compatible with all of them, this protocol must consider all available UAV flight times, including those of professional, consumer, toy, and military type drones. To date, the MQ-C Gray Eagle, a military drone, has the longest flight time of 25 h [27]. Thus, it is crucial to have efficient key management that all UAVs can support throughout their flight navigation.

**Figure 1:**
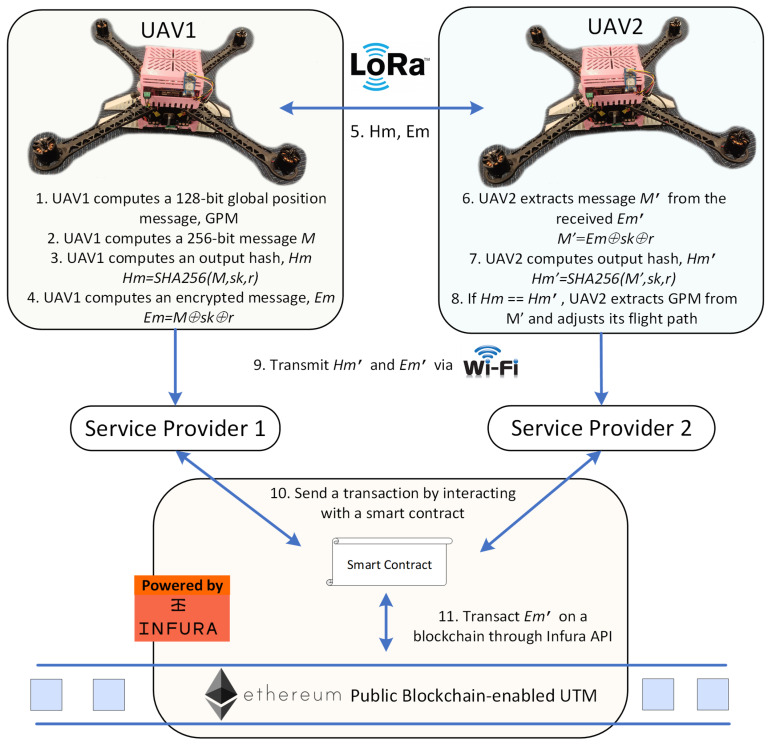
LoRa D2D Communication Protocol Using Public Blockchain-based UTM.

The proposed protocol consists of two phases: the initial phase and the authentication phase. Notations used in the protocol are listed in Table 1, and the infographic is depicted in Figure 1. The proposed protocol integrates a public blockchain to enhance transparency in the UTM system without relying on a central authority. The data stored on the public blockchain is immutable due to its decentralized ledger architecture, where each block is cryptographically linked to the previous one, ensuring tamper resistance and data integrity. The proposed protocol utilizes the SHA256 hash function to ensure the integrity of flight data by generating a unique and irreversible digest for each message, preventing any tampering. Additionally, the protocol uses bitwise XOR operations with a unique session key to encrypt flight data, protecting against security attacks while maintaining low computational overhead due to its lightweight nature.

### 3.1. Initial Phase

The initial phase consists of steps to be performed before the authentication phase, including session key generation. These steps are as follows:

1. Each service provider registers with a public blockchain-based UTM to obtain a pair of public and private keys, which can be used to make flight operation transactions on the Ethereum blockchain.
2. The UTM generates two random numbers, namely r and s, which will be returned to service providers.
3. The service providers generate two session keys, each being valid for a 24 h period in sequence to support flight operations that span more than one day (e.g., ${sk}_{1}$ is valid from 00:01 to 24:00 on the first day and ${sk}_{2}$ is valid from 00:01 to 24:00 on the second day). This session key, ${sk}_{1}$, or both session keys (${sk}_{1}$ and ${sk}_{2}$) as well as the random number, r, will be stored on the UAVs depending on their registered flight time.
  $$sk_{1}=SHA256\left(r,s,\;t_{day1}\right)\;$$
  $$sk_{2}=SHA256\left(r,s,\;t_{day2}\right)\;$$
  where $t_{day1}$ is the Unix timestamp of the start of the first day and $t_{day2}$ is the Unix timestamp of the start of the second day, as illustrated in Table 2.
4. The UAV operators register their UAVs with a service provider and obtain unique UAV IDs.
5. The UAV operators submit UAVs flight plans to service providers.
6. The service providers provide flight activities of UAVs to UTM, including the intended flight destination, flight path, and speed, by making transactions on the Ethereum public blockchain.

### 3.2. Authentication Phase

**Figure 2:**
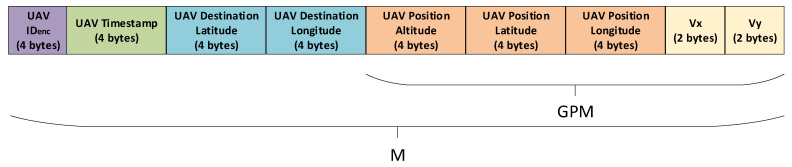
Structure of message M.

The authentication phase is initiated only when a received LoRa signal exceeds a predefined Received Signal Strength Indicator (RSSI) threshold (i.e., >−91 dBm). This threshold corresponds approximately to a distance of 1 km under line-of-sight conditions, indicating that the transmitting UAV is within proximity [28]. This mechanism significantly reduces the overall number of transmissions by ensuring UAVs only attempt authentication when they are in close proximity. Additionally, the transmission payload is limited to just 512 bits, which significantly reduces the ToA. For instance, using a 125 kHz bandwidth and a coding rate of 4/5, the ToA for a 512-bit payload is approximately 131 ms [29], well below the 400 ms of regional dwell-time restrictions defined in the AS923 band [30]. While LoRaWAN-specific limitations do not bind the proposed solution, it remains compliant with the underlying regional regulatory limits for the ISM band, including 1% duty cycle constraints [31], which permit up to 36 s of transmission time per hour. This equates to approximately 227 packets of 512 bits per hour. By reducing transmission frequency through proximity-based triggering and minimizing airtime with short payloads, the proposed solution ensures compliance with temporal occupancy regulations in the ISM band. Although the authentication phase is described using a two-UAV scenario, as shown in Figure 1, the proposed protocol is inherently scalable to multi-UAV environments. The proximity-based mechanism helps manage communication efficiently and reduce channel contention in densely populated networks. The following describes the steps needed for two UAVs, namely UAV1 and UAV2, to conduct the authentication phase during D2D communication, which are as follows:

1. UAV1 determines its global position via installed sensors, as shown in Table 3. It measures its velocity (i.e., vx and vy) by using an Inertial Measurement Unit (IMU). Velocity of vz is not included in the computed message M, since it is needed for take-off and landing and is not as vital as vx and vy, which are critical for navigation and maintaining UAV flights. It also obtains its longitude, latitude, and altitude using the Global Positioning System (GPS). These data will be concatenated to a 128-bit global position message, GPM, as described in Table 3.
2. UAV1 then encrypts the 32-bit UAV ID by XOR-ing it with the 32-bit Unix timestamp, where ${ID}_{enc}=ID⊕t$. It then concatenates the 32-bit UAV ${ID}_{enc}$, current 32-bit Unix timestamp, t, a 64-bit UAV flight destination, and the 128-bit global position message, GPM, to construct a 256-bit concatenated message, M, as presented in Figure 2.
3. UAV1 then computes an output hash, Hm, by hashing M with the session key, sk, where Hm=SHA256(M,sk, r). This output hash is used for data integrity protection purposes.
4. UAV1 computes an encrypted message, Em, by bitwise XOR-ing M with sk and r, where Em=M⊕sk⊕r.
5. UAV1 broadcasts the output hash, Hm, and the encrypted message, Em, to nearby UAVs through LoRa peer-to-peer communication to update its global position.
6. UAV2 receives the $Hm^{\prime }$ and $Em^{\prime }$ values from UAV1. The UAV2 then extracts the concatenated message, M, from the Em′ by XOR-ing Em′ with its stored sk and r, where $M^{\prime }=Em^{\prime }⊕sk⊕r$.
7. UAV2 proceeds to compute an output hash, Hm′, by performing hashing of $M^{\prime },\;sk,$ and r using the SHA256 hash function, where $Hm\prime =SHA256(M^{\prime },\;sk,\;r)$.
8. UAV2 authenticates the messages sent from UAV1 if the computed output hash, Hm′, is equal to the received output hash, Hm. UAV2 then extracts the global position data from the concatenated message, M′, and adjusts its flight path if there is a potential collision with the nearby UAV1. Otherwise, the UAV2 terminates the session and deletes the received message, M.
9. UAVs transmit the output hash, Hm′, and the encrypted Em′ to service providers when they are covered by Wi-Fi. The authentication of the UAVs will be verified, and the message, M, will be decrypted by the service providers using their stored sk and r.
10. The service providers update the global position of UAVs by making transactions of encrypted Em′ on the Ethereum public blockchain. The UAVs flight activities can only be decrypted and viewed by authorized service providers.

The same steps apply to the communication initiated by UAV2 with UAV1 and other nearby UAVs.

## 4. Proof of Concept

### 4.1. Development of UAVs with an Onboard LoRa Communication Module

**Figure 3:**
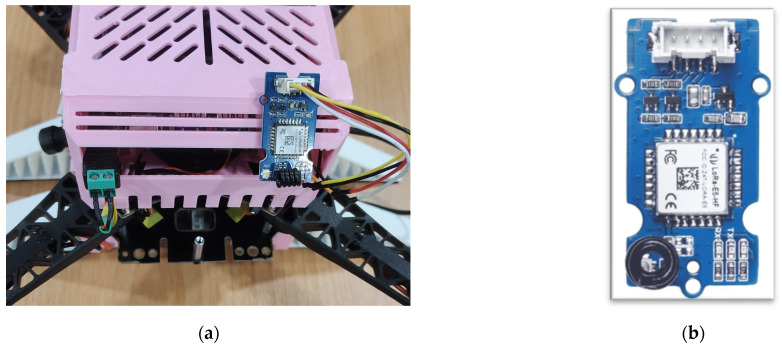
(**a**) A LoRa node attached to an S500 UAV; (**b**) Grove LoRa E5 node.

An in-house UAV equipped with a Grove LoRa-E5 module (Seeed Studio, Shenzhen, China) was used for the proof of concept, as shown in Figure 3. The module supports LoRa communication and has an integrated ARM Cortex-M4 (ARM Holdings, Cambridge, UK) ultra-low-power Microcontroller Unit (MCU), specifically the STM32WLE5JC (STMicroelectronics, Geneva, Switzerland). The integrated LoRa SX126x transceiver (Semtech, Camarillo, CA, USA) is configured to operate at 923 MHz. The UAV is built using the S500 Quadrotor kit (Holybro, Shenzhen, China) and features an onboard Jetson Orin Nano computer (Seeed Studio, Shenzhen, China) for real-time processing of deep learning and computer-vision tasks. It also features a Pixhawk 6C Mini flight controller (Holybro, Shenzhen, China) with a custom firmware based on the PX4 v1.15 version.

### 4.2. Development of a Decentralized UAV Traffic Management Web Application Platform

A decentralized UAV traffic management web application platform was created to enable each service provider to view UAVs flight activities through a public blockchain. The Ethereum Sepolia testnet was used for this purpose. A public blockchain is transparent, which enables all users, including attackers, to view the transactions, including their inputs. Hence, it is necessary to encrypt the transaction inputs to allow authorized service providers to view the UAV flight activities.

In order to achieve this, a smart contract was designed to store encrypted flight information on the blockchain using an *updateFlight*() function, as described in Algorithm 1. This function is restricted to the contract owner through the use of the *onlyOwner*. This access control mechanism prevents unauthorized users from modifying the state of the contract, thereby preventing attackers from exploiting the function through front-running attacks.

**Figure 4:**
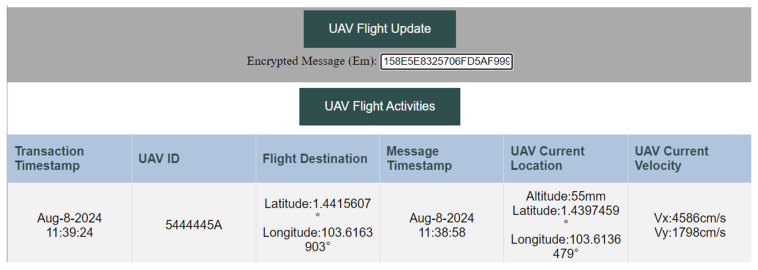
Decentralized Web Application for a Public Blockchain-based UTM.

The decentralized web page was designed using HyperText Markup Language (HTML), and JavaScript was used to enable interactive content on the webpage. To interact with the Ethereum Sepolia testnet and smart contracts, the Web3.js library is used. In addition, Infura was used to interact with the testnet by accessing Ethereum remote nodes. This platform enables authorized service providers with correct r and sk values to decrypt the transaction inputs and view the flight details, as shown in Figure 4. The UAV ID is obtained by XOR-ing the ${ID}_{enc}$ extracted from the message *M* with the extracted timestamp, t. Attackers will only be able to obtain encrypted input from transactions and will be unable to decrypt to obtain the correct flight details.
**Algorithm 1:** Pseudo-code of *updateFlight()***Input:** valueEm: bytes321. if (msg.sender == owner) 2. if (balance > transaction fee) 3. assign valueEm to storedEm 4. else 5. revert the transaction 6. end 7. else 8. revert the transaction 9. end

## 5. General Security Analysis

### 5.1. Secret Key Disclosure Attack

Attackers may attempt to eavesdrop on the communication and obtain transmitted messages. However, no plaintext is transmitted, only an output hash, Hm, and an encrypted message, Em are broadcasted. In addition, the secret key, sk, is computed by hashing random numbers r and s, as well as a timestamp. Crucially, the random number s is never disclosed to the UAVs operators or transmitted over the network. Since the SHA256 hash function is resistant to preimage attacks [32], the attackers are unable to reverse engineer the session key, even with knowledge of the timestamp, because the secret random number s is used. Thus, the attackers are unable to extract the secret session key, sk or the underlying random numbers, r and s from intercepted messages.

In addition, although the blockchain stores the encrypted message, Em, decryption is only possible with the correct r and session key sk. The session key, sk, is generated using the SHA256 function, based on a secret random number s, which remains undisclosed. Since the blockchain is publicly visible, attackers may attempt to scan historical data for potential decryption attempts in the long term. Nonetheless, due to the strong preimage resistance of SHA256, successful preimage attacks are not expected to be feasible for at least the next decade [33]. This preserves forward secrecy and prevents disclosure of the secret key.

### 5.2. Replay Attack

If attackers replay previously transmitted messages to nearby UAVs, this attack will be unsuccessful due to the inclusion of the current timestamp in the transmitted messages. UAVs receiving the transmitted message will verify the extracted timestamp with the current one. The LoRa transmission time in the worst-case scenario, with a bandwidth of 125 kHz and a Spreading Factor (SF) of 12, is 4.245 s [34]. Thus, if the timestamp difference exceeds 10 s, the authentication of legitimate D2D communication will fail, and the nearby UAVs will not further process the message. However, dynamic conditions, such as congestion and varying UAV density, may introduce transmission latency, potentially leading to false detection of replay attacks. This will be addressed in future work by implementing an adaptive time window adjustment to calibrate the allowable timestamp difference dynamically. The Receive Signal Strength Indicator (RSSI) will be used as a real-time metric for assessing network congestion.

Furthermore, this design mitigates length extension attacks, which typically allow an attacker to create a valid hash by appending data to a partially known input. This protocol generates a unique session key for each communication session using a hash of a random number r, a secret random number s, and the current timestamp. As explained in Section 5.1, attackers are unable to extract the session key from transmitted messages because the secret value is never transmitted or disclosed. The attackers are unable to decrypt the message and reconstruct the original plaintext without access to the session key, making it impossible to append data for a length extension attack. Additionally, these attacks will not succeed, as the protocol is resistant to replay attacks through the use of unique session keys and timestamp validation.

### 5.3. Adversary-in-the-Middle Attack

Attackers might attempt to eavesdrop, block, and modify transmitted messages, including Hm and Em. However, these attacks will be unsuccessful because attackers cannot generate the same message, M, using the modified Hm′, and Em′. Therefore, if the computed hash, Hm differs from the received hash value, Hm′, peer UAVs will reject the modified message. Furthermore, even if attackers intercept multiple encrypted messages and know the format of M, they are unable to extract the session key, sk, or the random numbers, r and s. This is because the secret random number s is used in the key generation and is never disclosed or transmitted. As a result, the protocol is resistant to known plaintext attacks and remains secure under adversarial conditions.

### 5.4. Tracking Attack

Attackers are unable to trace transmitted messages, Hm and Em, as these messages vary for each communication session. Message M consists of the concatenation of the encrypted UAV ${ID}_{enc}$, timestamp, and UAV information, which vary for each session. In addition, message M is encrypted with a session key, sk, and a random number, r. Thus, the attackers are unable to extract the UAV ID from the message, Em, and are unable to trace any specific UAV from the communicated messages.

## 6. Comparative Performance Analysis

As mentioned in Section 2, no research work was found on designing secure D2D LoRa communication for public blockchain-based UTM systems. Therefore, this section analyzes the performance of the proposed D2D LoRa communication protocol in terms of security, computation, storage, and payload size. Ethereum blockchain and SHA256 hash function security analyses are not included in this paper as they have already been proven secure [35,36].

### 6.1. UTM Access Management with D2D Communication Integration

Blockchain technology has been proposed for integration with UTM to enable a secure and efficient traffic monitoring system through controlled access to flight data [7,9]. Alkadi and Shoufan implemented access control modifiers for each function in smart contracts to manage the privileges and authorities of users on a permissioned public blockchain [9]. Different Ethereum addresses are used to represent various roles, such as UAV operator, public reporter, authority smart contract, and UAV service provider. However, this approach increases the complexity of the UTM system, as keeping track of the addresses and role assignments is challenging and creates scalability issues.

Allouch et al. proposed to use the Hyperledger Fabric, a private blockchain, for identity management and access control [7]. This approach allows nodes to control and limit access to their distributed sensitive information. UAVs must constantly update their ground control stations with flight information. The data are then sent to the cloud server, encrypted, and stored in an off-chain database (i.e., OrbitDB with IPFS). The data are subsequently hashed and stored as an immutable transaction on the blockchain. However, a private blockchain is not fully decentralized and, hence, susceptible to security attacks.

To ensure transparency and enhanced security, this paper proposes developing a UTM system using a public Ethereum blockchain, allowing users to view transactions while only authorized service providers with legitimate secret keys can view flight data. Communication latency is the main issue in [7,9] because it does not support real-time D2D communications, which is critical for adapting UAVs flight paths to avoid obstacles. The proposed solution addresses this by supporting direct D2D communication, enabling more efficient collision avoidance for UAVs. The message transmitted during D2D communication will be updated on the Ethereum blockchain in the proposed UTM system for better flight monitoring.

### 6.2. Computation Cost

The proposed protocol utilizes one SHA256 function to generate a 256-bit Hm. It further uses one bitwise XOR operation to encrypt the UAVs’ ID, followed by two additional bitwise XOR operations to compute a 256-bit encrypted message, Em. The sensitive flight information in message M is encrypted to prevent security attacks. The computational cost has been analyzed and compared with related works where various IoT devices, ranging from resource-constrained IoT devices like the Arduino Uno to more powerful devices, such as the Galaxy S5 smartphone (Samsung, Suwon, Republic of Korea), have been used. The companion board used for the proof of concept is the Jetson Orin Nano, a device with 8 GB of RAM and a 1.5 GHz CPU clock speed. The average computation time for a SHA256 function and three XOR operations using the aforementioned hardware is approximately 0.01 ms.

**Figure 5:**
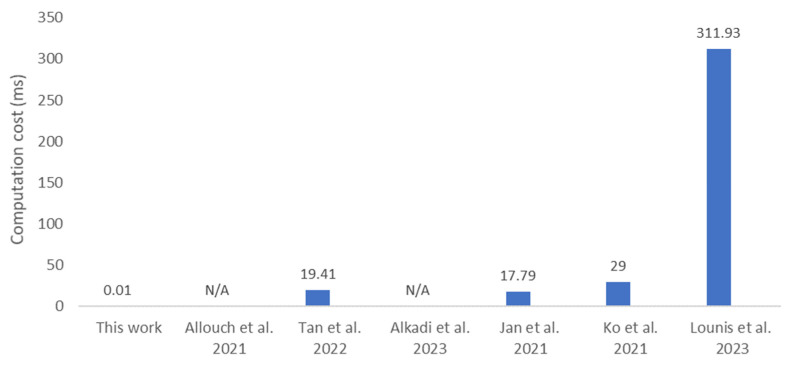
Comparative computation cost [7,8,9,24,25,26].

In addition to the computation cost, for a fair comparison with the related studies, the cryptography used in the protocols is analyzed. Figure 5 illustrates that the proposed protocol has the lowest computation cost compared to related works, due to its lightweight design, which requires only 1 SHA256 and 3 XOR operations. In contrast, the protocol in [25] takes 29.00 ms, as it involves performing both the Elliptic Curve Digital Signature Algorithm (ECDSA) and Hash-based Message Authentication Code (HMAC). The solution in [26] involves 16 hash function operations and 10 bitwise XOR operations, requiring a computation time of 311.93 ms. The protocol in [24] requires 17.79 ms for authentication, involves one multiplication, seven HMAC, and eight XOR operations, while [8] requires 19.41 ms to perform Elliptic Curve Cryptography (ECC). Some other related works transmit flight data as plaintext, eliminating the need for additional computation processes. According to Big-O notation, the computation cost in [9] addresses only the implementation of smart contracts, which is O(1) in complexity, as is the case in [7].

### 6.3. Storage Cost

**Figure 6:**
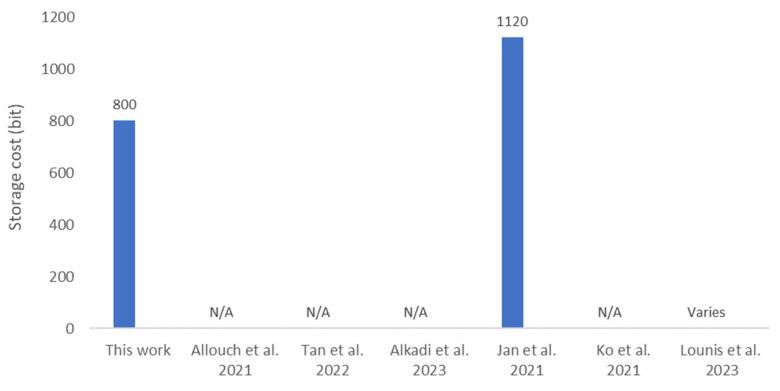
Comparative storage cost [7,8,9,24,25,26]. N/A: Data Not Available at the source.

The storage cost analysis of the proposed protocol is focused solely on UAVs, as service providers and UTM systems can support more resource-intensive computations. UAVs are required to store either a 256-bit session key, ${sk}_{1}$, or both session keys (${sk}_{1}$ and ${sk}_{2}$), depending on their flight duration, as described in Section 3. Additionally, UAVs must also store a 256-bit random number, r, and a 32-bit UAV ID. Thus, the total storage cost for UAVs ranges from 544 bits to 800 bits, based on the flight duration, which is manageable even for resource-constrained UAVs. Figure 6 shows that the storage cost for the proposed protocol is the lowest compared to related works, as [24] requires 1120 bits. Although [26] uses a Physical Unclonable Function (PUF) for credential generation, the protocol requires a storage cost that varies with the number of registered UAVs (*n*), specifically 2*n* + 3 for a 64-bit configuration. Thus, the storage cost becomes significantly high and unsupported by resource-constrained UAVs when the number of registered UAVs becomes large.

### 6.4. Formal Security Analysis

Formal security analysis is important in addition to the general security one, since it provides a rigorous method to prove the correctness of the proposed protocol. Although the protocol proposed in [8] has not undergone formal security analysis, other related works have been analyzed using formal methods, including modal-logic-based analysis [25] or verification tools [24,25,26].

The proposed protocol is modeled using the High-Level Protocol Specification Language (HLPSL) and analyzed with a widely used formal security verification tool, AVISPA. This security verification tool has four back-end verification engines. The On-the-Fly Model Checker (OFMC) back-end was selected for analyzing the proposed protocol because it effectively checks for security vulnerabilities that this paper aims to examine. The security flaws include adversary-in-the-middle attacks, replay attacks, tracking attacks through state space exploration, key disclosure attacks through symbolic analysis, and authentication flaws.

**Figure 7:**
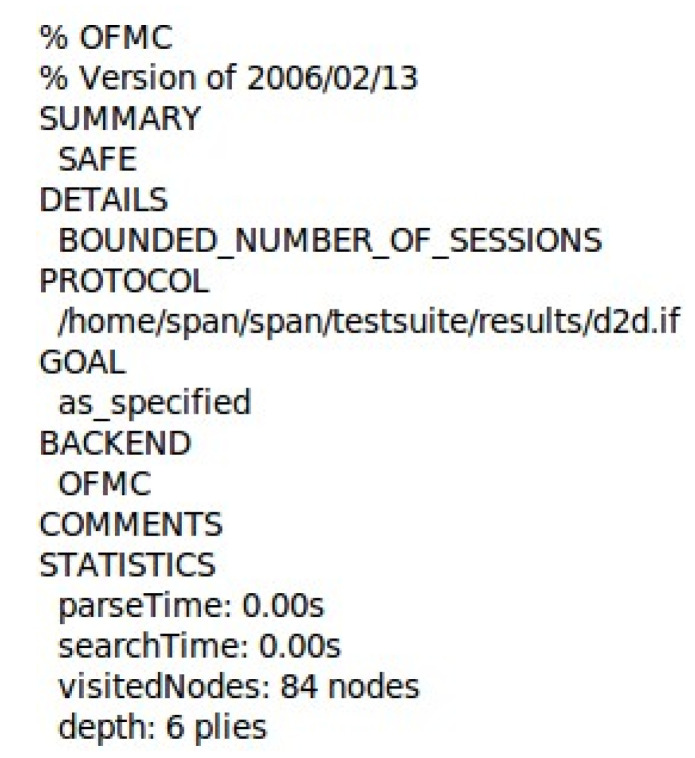
OFMC Security Result.

Figure 7 shows that the OFMC used an extremely small and unmeasurable amount of time (0.00 s) to read and process the input specification of the protocol. The search process of OFMC is efficient, as it took the same amount of time to explore the state space for vulnerabilities. The OFMC result shows that the proposed protocol is safe from the aforementioned attack, having been visited by 84 states and explored up to six levels of the protocol.

### 6.5. LoRa Payload Size Analysis

Most related works do not include hardware implementations of communication technologies [7,8], and some barely mention that UAV relies on wireless technologies [24,25,26]. While [9] specifically mentions the use of Wi-Fi and Bluetooth, it lacks the analysis of communication performance. This paper proposes using LoRa technology in D2D communication to achieve wider signal coverage. Although LoRaWAN communication has payload size limits for both uplink and downlink transmission through the gateway based on data rate and SF [30], these constraints do not apply in LoRa. Furthermore, while high SF allows for greater sensitivity and longer transmission range, it requires longer transmission times, directly increasing the power consumption. Therefore, SF_7_ to SF_9_ have been identified as the most suitable options for achieving balanced communication in IoT networks, optimizing both transmission range and power consumption [37].

This paper utilizes LoRa peer-to-peer communication, making it essential to determine the payload size limitation for message transmission without relying on gateways. Table 4 presents experimental results for transmitting 512-bit messages, consisting of 256 bits of Hm and 256 bits of Em, across a range of SFs from 7 to 12, independent of the LoRaWAN payload size limit discussed in [30]. In addition to bypassing payload size constraints, the proposed LoRa peer-to-peer solution does not enforce LoRaWAN-specific MAC layer constraints outlined in [30], such as the join duty cycle, Receive Window 1 Data Rate Offset (RX1DROffset), dwell time restriction, and band-specific limitations. This flexibility allows for the use of a wider range of SFs, enabling the selection that provides sufficient coverage for typical D2D communication distances. Under line-of-sight conditions, LoRa performance demonstrates that SF_7_ can support ranges of approximately 2 km, while SF_12_ can extend coverage up to 14 km in open environments [38]. However, these distances may vary depending on environmental factors, especially in scenarios involving densely populated UAV networks, which will be further analyzed in future work.

### 6.6. Smart Contract Vulnerability Analysis

Although smart contracts have been used in works [7,8,9], only [9] analyzes smart contract vulnerability using verification tools, including SmartCheck, Oyente, Osiris, and Slither. Thus, smart contracts deployed in [7,8] may be susceptible to vulnerabilities due to the lack of thorough analysis and verification.

**Figure 8:**
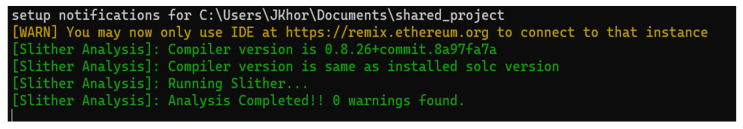
Slither Analysis Result.

A well-accepted static code verification tool, Slither, has been used to analyze the d2d.sol for potential vulnerabilities, including shadowing, uninitialized variables, and reentrancy. Slither generates an Abstract Syntax Tree (AST) from the d2d.sol and then transforms it into an internal representation language, SlithIR, to compute various code analyses [39]. Figure 8 shows the Slither analysis result with no errors or warnings found in the *d2d.sol*.

Table 5 provides a comparative performance summary of the proposed solution against related works. The results demonstrate that none of the existing studies have integrated D2D communication with a UTM system, a feature that is uniquely addressed by this solution. In addition, the proposed D2D protocol exhibits the lowest computation and storage costs, supported by a comprehensive general and formal security analysis, as well as a detailed performance evaluation of the overall system.

## 7. Limitations and Future Work

A key limitation of the current system is the use of a static RSSI threshold to estimate UAV proximity. In practice, environmental factors such as interference, multipath fading, and antenna orientation may cause RSSI values to fluctuate, leading to false proximity detection or missed communication opportunities. Moreover, dynamic network conditions, such as UAV density and channel congestion, can introduce transmission latency, potentially leading to the false detection of replay attacks.

To address these limitations, future work will focus on enhancing the proximity detection mechanism by incorporating adaptive RSSI thresholds, Signal-to-Noise Ratio (SNR) values, and GPS-based distance estimation. An adaptive window calibration mechanism will also be introduced to account for latency variations. Furthermore, RSSI will be utilized as a real-time metric to assess network congestion and guide the dynamic adjustment of protocol parameters. A comprehensive scalability analysis will be conducted through simulations and testbed evaluations to quantify the protocol’s performance in terms of latency, collision rate, and throughput under different UAV densities.

## 8. Conclusions

This paper addresses a significant research gap in UTM studies by integrating secure direct D2D communication to avoid collisions efficiently. The proposed D2D protocol supports a wide range of UAVs, regardless of their flight duration or service provider. A public Ethereum blockchain is utilized to develop a secure UTM, ensuring that the sensitive flight data are accessible only to the authorized service providers with the appropriate secret keys. A proof of concept demonstrates the feasibility of this UTM system, and the proposed D2D protocol has been rigorously evaluated by means of a general security analysis, with its formal security properties being verified by the AVISPA tool. It has been proven to be secure against key disclosure, adversary-in-the-middle, replay, and tracking attacks. Performance evaluation shows the protocol achieves the lowest computation and storage costs, at 0.01 ms and 544–800 bits, respectively. The feasibility of using LoRa technology for D2D communication is also validated, with a successful message transmission across all SFs despite payload size limitations. Future work will focus on improving proximity detection and implementing an adaptive time window adjustment to dynamically calibrate allowable timestamp differences. The scalability of the proposed system will further be evaluated by analyzing UAV performance under varying density conditions.

## Figures and Tables

**Table 1:** Notation Used in The Proposed Protocol.

Notation	Description	Purpose
ID	A 32-bit UAV ID	To identify the UAV identity
${ID}_{enc}$	A 32-bit encrypted UAV ID	To prevent tracking attacks on UAV identity
r	A 256-bit random number	To provide a random value for hash output and encryption
s	A 256-bit random number	To be used for session key updates
$t_{day1}$	A 32-bit Unix timestamp for the start of the first day	To be used for the generation of *sk*_1_
$t_{day2}$	A 32-bit Unix timestamp for the start of the second day	To be used for the generation of *sk*_2_
t	A 32-bit current Unix timestamp	To be used for the generation of message M
${sk}_{1}$	A 256-bit session key for the first day	Session key for UAVs for flight time within a day
${sk}_{2}$	A 256-bit session key for the second day	Additional session key for UAVs for flight time of more than a day
SHA256	SHA256 hash function	To generate a 256-bit hash output
⊕	Bitwise XOR	To encrypt a flight message
GPM	A 128-bit global position message	UAV flight information
M	A 256-bit concatenated message	A concatenation of a Unix timestamp and a global position message
Hm	A 256-bit output hash	To ensure the data integrity of message M
Em	A 256-bit encrypted message	To safeguard message M from security attacks

**Table 2:** Example of Unix Timestamp Used in Session Key Generation.

Nonce	Start of Day	Unix Timestamp
$t_{day1}$	20 July 2024, 12:00:00 a.m.	1721433600
$t_{day2}$	21 July 2024, 12:00:00 a.m.	1721520000

**Table 3:** Global Position Message Detail.

Field	Description	Data Type	Units
alt	Altitude	Int32	mm
lat	Latitude	Int32	degE7
lon	Longitude	Int32	degE7
vx	Ground X speed	Int16	cm/s
vy	Ground Y speed	Int16	cm/s

**Table 4:** LoRa Payload Length Limit.

Data Rate (DR)	Spreading Factor (SF)	Channel Frequency (kHz)	AS923 Payload Size (Bytes)	Successful Message Transmission Using LoRa Peer-to-Peer
0	SF_12_	125	51	Yes
1	SF_11_	125	51	Yes
2	SF_10_	125	51	Yes
3	SF_9_	125	115	Yes
4	SF_8_	125	242	Yes
5	SF_7_	125	242	Yes
6	SF_7_	250	242	Yes

**Table 5:** Comparative Performance Analysis.

Description	This Work	[7]	[8]	[9]	[24]	[25]	[26]
Support UTM	Yes	Yes	No	Yes	No	No	No
Blockchain	Ethereum	Hyperledger Fabric	Hyperledger Fabric	Ethereum	N/A	N/A	N/A
Blockchain type	Public	Permissioned private	Permissioned consortium	Permissioned public	N/A	N/A	N/A
Access control	Symmetric key	Private blockchain	Consortium blockchain	Function access modifier	N/A	N/A	N/A
Support D2D communication	Yes	No	Yes	No	Yes	Yes	Yes
IoT device used for proof of concept	Arduino Uno R3	N/A	Windows 10 PC	N/A	Samsung Galaxy S5	Raspberry Pi	Arduino Uno
Computation cost (ms)	0.01	N/A	19.41	N/A	17.79	29.00	311.93
Cryptography used in D2D protocol	1 SHA256 3 XOR	N/A	ECC	N/A	1 Mult. 7 HMAC 8 XOR	ECDSA 7 HMAC	16 Hash Funct. 10 XOR
Storage cost (bit)	544–800	N/A	N/A	N/A	1120	N/A	* Varies
Encrypted transmitted message	Yes	No	Yes	No	No	Yes	No
**Security analysis of D2D communication** Protection against:							
Secret key disclosure attack	Yes	N/A	Yes	N/A	Yes	Yes	Yes
Adversary-in-the-middle attack	Yes	N/A	Yes	N/A	Yes	Yes	Yes
Replay attack	Yes	N/A	Yes	N/A	Yes	Yes	Yes
Tracking attack	Yes	N/A	No	N/A	Yes	Yes	No
Drone communication technology	LoRa	N/A	N/A	Wi-Fi Bluetooth	Wireless tech.	Wireless tech.	Wireless tech.
Experimental analysis of drone communication	Yes	N/A	N/A	No	N/A	N/A	N/A
Smart contract vulnerability analysis	Yes	No	No	Yes	N/A	N/A	N/A

* Depends on the number of registered UAVs. N/A: Data Not Available at the source.

## Data Availability

Relevant data are contained within the article.
